# The Prevalence of Depression and Related Factors During the COVID-19 Pandemic Among the General Population of the Jazan Region of Saudi Arabia

**DOI:** 10.7759/cureus.21965

**Published:** 2022-02-06

**Authors:** Abdullah Alharbi

**Affiliations:** 1 Family and Community Medicine Department, Faculty of Medicine, Jazan University, Jazan, SAU

**Keywords:** saudi arabia, psychological effect, public health, depression, covid-19

## Abstract

Background

This study examines the rates of depression associated with the COVID-19 pandemic along with mitigation measures such as lockdown and quarantine in the population of the Jazan region in Saudi Arabia. The Kingdom of Saudi Arabia (KSA) began mitigation measures before the first case appeared on March 2, 2020, disrupting daily life in a culture that is centered on family life. We sought to assess the psychological impacts of the pandemic on this culturally unique region to see if it affected as many as other reported places in the world.

Methods

A self-reporting online questionnaire in Arabic was distributed through social media applications and a convenience sample of 942 participants ≥18 years of age living in the Jazan region was selected. The questionnaire included socio-demographics, economic status, chronic medical conditions, focus on and knowledge of COVID-19, and the patient health questionnaire-9 scale (PHQ-9) for depression metrics.

The data in this study were analyzed using descriptive analysis of participant characteristics, followed by Chi-square testing to compare reported depression related to each variable. Finally, to control for confounding factors, we applied multivariate logistic regression to find an adjusted odds ratio (AOR) with a 95% CI.

Results

In the Jazan region, the rate of depression during the COVID-19 pandemic was nearly 26%. There are several significant determinants associated with higher rates of depression in descending order: those with chronic diseases were 160% higher than those without; those with a history of mental illness were 150% higher; participants who focused excessively on the pandemic ≥3 hours daily were 130% higher; participants who were divorced or widowed were 120% higher than singles; females were 87% higher; those under age 40 were 57% higher; students were 50% higher; those reporting low incomes were 40% higher than those with moderate incomes and 60% higher than those with high incomes.

Conclusions

Strategies need to be devised to protect vulnerable groups of participants from mental health effects, including depression during the COVID-19 pandemic. This will require the collaboration of various institutions, such as schools and others, to provide support for education and mental health. Future research should be aimed at determining the reasons for this higher vulnerability of some groups.

## Introduction

In January 2020, a novel coronavirus identified as SARS-CoV 2 spread rapidly around the world from its initial discovery in Wuhan, China, and the pandemic disease it causes, COVID-19, has continued for nearly two years [1]. The first case occurring in the Kingdom of Saudi Arabia (KSA) was reported on March 2, 2020, after the KSA had begun mitigation measures [2,3]. Mitigation measures were implemented in stages by the KSA and included requirements that radically changed the Saudi culture, which has close social daily interaction with family. Some examples of the stressful restrictions put in place were the closing of domestic travel, including to the holy cities of Mecca and Medina, as well as curfews that were imposed to prevent citizens from leaving their homes except during certain hours with a limit of only one person per household permitted to go out to obtain food and necessary supplies for the household [4,5]. The suspension of school and work, along with the enforcement of shelter at home policies, presented both a psychological and economic burden on Saudi daily life. While the physical medical care of acute COVID-19 is necessary, as one of the top 25 global illnesses of concern, depressive disorder is costly for individual wellbeing as well as society and the economy, and is a significant factor in suicide, as revealed by a review by Santomauro [6]. Symptoms of mental health disorders such as depression, anxiety, OCD, suicide, neurological, cognitive, and others have been increasing worldwide during the COVID-19 pandemic as the medical, economic, and psychological burdens continued [7-10]. These effects have proven to be long-lasting in previous pandemics such as MERS and SARS [9]. Therefore, this study is critical to assess their prevalence in the Jazan region and formulate plans for strengthening mental health along with medical conditions caused by COVID-19.

Many international studies have reported increased depression rates during the COVID-19 pandemic. Two U.S. studies comparing COVID-19-related depression to pre-covid rates on 18- to 30-year-olds observed an increase in depression following the COVID-19 shutdown of three-sevenfold, with the main factors being fewer socio-economic resources, the sudden high rate of unemployment, loneliness, and uncertainty about the course of the pandemic, while family support was very important in protecting participants from depression [7,8]. A study in Italy of the mental health of COVID-19 survivors revealed high levels of depression, possibly related to immune responses to the disease, along with some long-lasting disabilities [11]. A large systematic review and meta-analysis encompassing more than 33,000 subjects from 12 Chinese studies and one from Singapore on healthcare workers was conducted by scholars in the UK and Greece, revealing an overall depression rate of 22.8%, with the highest rates among females and nurses [12]. Investigators in Turkey examined depression, anxiety, and health anxiety during the COVID-19 period and found the rate of depression to be slightly more than 23%, with the strongest correlating factor being the female gender [13]. Chinese scholars examined depression and anxiety using an online questionnaire distributed nationally to adults who did not have a previous history of mental health conditions and reported a 20.4% rate of depression and/or anxiety or both, with a relationship to time spent focusing on COVID-19 news [14].

There are several Saudi studies of depression rates in the KSA related to COVID-19. As with international studies, multiple authors found similar factors contributing to symptoms of depression, such as being female, being younger than 30 years old, spending a lot of time focused on COVID-19 news, and having someone with COVID-19 within their circle of acquaintances [15-18]. Studies conducted on healthcare workers in the KSA and Egypt and university students in the KSA revealed depression rates of 69% and nearly 49%, respectively, with additional related factors of lack of emotional support and the presence of pre-existing medical or mental conditions [15,18]. Other studies cited other factors that are associated with increased rates of depression, such as being non-Saudi, unemployment, low income, being over 50 years old, being divorced, being retired, being single, and smoking [19,20]. Among the studies of the general population, depression rates were observed to range from 9.4% to 65%, but none of these studies concentrated on a single region, such as the Jazan region [16,17,19].

The aim of this study was to examine COVID-19-related depression rates and associated factors for the Jazan region of the KSA in order to provide background for planning purposes. Any pandemic will have an impact on the economic conditions and psychological health as well as the physical health of a population. Therefore, it is prudent to assess the experience of the COVID-19 pandemic to provide awareness of the importance that mental health plays in the resilience of society. This study provides guidance to both address the current status of psychological health in the population of the Jazan region as well as prepare for future traumatic events with the goal of prevention of the worst effects.

## Materials and methods

Study procedure

An electronic questionnaire was created in Arabic using the Google Survey tool (Google LLC, Mountain View, California, USA) to conduct a cross-sectionally designed study to assess depression levels related to the COVID-19 pandemic and quarantine in the Jazan region of the KSA. The survey was then distributed via the social media platforms Twitter^©^ and WhatsApp^©^ and users were invited to participate if they met the inclusion criteria during the four-day period of May 28, 2020 through May 31, 2020. A convenience sample was selected from the eligible respondents. This self-reporting survey included sections on demographics, socioeconomic characteristics, pre-existing medical and mental health conditions, and the patient health questionnaire-9 scale (PHQ-9), an Arabic version whose validity has been established by Cronbach’s α = 0.857, with questions about depressive symptoms [21]. An online survey was necessary during the period of the COVID-19 pandemic lockdown since it was not possible to conduct in-person surveys. This method is in widespread use by researchers around the world [8,17-20]. The survey, titled "Psychological Impact(s) in Southern Saudi Arabia" was accessible via a link that explained the following inclusion criteria: participants had to be ≥18 years of age, residents of the Jazan region of the KSA, able to read and understand Arabic, and physically, mentally, and emotionally able to complete the form. Exclusion criteria included being <18 years of age, not being a resident of the Jazan region, being a non-Arabic speaker, and being mentally, emotionally, or physically unable to answer questions. The survey cover page explained the purpose of the questionnaire and the consent.

Data collection

Data were collected for 942 participants who voluntarily filled out an electronic questionnaire distributed on May 28, 2020 through May 31, 2020 on either Twitter© or WhatsApp©. Participant inclusion criteria were ≥18 years old, currently living in the Jazan region of the KSA, Arabic speakers, physically capable of filling out the form, and mentally and emotionally able to fill out the form. Exclusion criteria were <18 years old, not currently living in the Jazan region of the KSA, non-Arabic speakers, and not physically, mentally, or emotionally able to fill out the form. Informed consent was included on the questionnaire, and the privacy of the participants was maintained throughout the data collection. The questionnaire was composed of four sections of questions on socioeconomics, demographics, focus on and knowledge of COVID-19, and mental health symptoms.

The Epi InfoTM7 (Centers for Disease Control and Prevention, Atlanta, Georgia, USA) was employed to determine a statistically sufficient sample size. The minimum required sample size calculated through this method was 511 in order to provide no more than a 5% margin of error with a 99% confidence interval (CI). Therefore, our sample size of 942 increased the power of our statistical model [22]. We anticipated that nearly doubling the sample size would avoid sampling bias with the use of an online questionnaire [22].

Data and measurements

Data were collected for 942 participants who voluntarily filled out an electronic questionnaire distributed on May 28, 2020 through May 31, 2020 on either Twitter^©^ or WhatsApp^©^. Participant inclusion criteria were ≥18 years old, currently living in the Jazan region of the KSA, Arabic speakers, physically capable of filling out the form, and mentally and emotionally able to fill out the form. Exclusion criteria were <18 years old, not currently living in the Jazan region of the KSA, non-Arabic speakers, and not physically, mentally, or emotionally able to fill out the form. Informed consent was included on the questionnaire, and the privacy of the participants was maintained throughout the data collection. The questionnaire was composed of four sections of questions on socioeconomics, demographics, focus on and knowledge of COVID-19, and mental health symptoms.

The Epi InfoTM7 (Centers for Disease Control and Prevention, Atlanta, Georgia, USA) was employed to determine a statistically sufficient sample size. The minimum required sample size calculated through this method was 511 in order to provide no more than a 5% margin of error with a 99% confidence interval (CI). Therefore, our sample size of 942 increased the power of our statistical model [22]. We anticipated that nearly doubling the sample size would avoid sampling bias with the use of an online questionnaire [23].

Statistical analysis

The Statistical Package for Stata 2014 version (StataCorp LLC, Texas, USA) was used to analyze all the data, with a p-value of 0.05 considered statistically significant. The data in this study were analyzed in a stepwise process. The first step was to perform a descriptive analysis of participant characteristics. The second step was to use Chi-square testing to compare reported depression related to each variable. In the third step to control for confounding factors, we applied multivariate logistic regression to find an adjusted odds ratio (AOR) with a 95% CI.

## Results

Table 1 presents a description of the characteristics of the participants grouped into those reporting and those not reporting depressive symptoms (DS). The following characteristics showed statistically significant differences: 86% of the overall sample was aged <40, and those under 40 accounted for more than double the number reporting DS. Females made up three-fourths of the total sample, and they accounted for one-and-a-half times as many among those reporting DS. Married participants represented only 40% of the sample and also had only one-fifth of those with reported DS, while divorced or widowed participants had double the reported DS. Of the total participants, less than one-fifth had chronic diseases, but they accounted for 60% more of the reported DS. Only 3% of the total sample reported previous mental illnesses, but they accounted for almost double the number of those reporting DS. Students reported one and a half times the rate of DS as those working and unemployed or retired, representing approximately 20% higher reported DS. In regards to the standard of living (SOL), those who reported a limited SOL and those with a high SOL each represented approximately 20% of the total sample. However, those with a limited SOL reported more than double the DS as those with a high SOL, while those reporting a moderate SOL fell between the two extremes. While 98% of participants reside with others, those participants reported five times the rate of DS. The final characteristic that showed statistical significance was time spent focusing on COVID-19, in which increasing time is directly related to reported DS, such that those spending ≥3 hours per day reported DS at a rate nearly double that of those who focused on it <1 hour per day. All other characteristics did not show statistically significant differences among those reporting DS. These include nationality, a diagnosis of COVID-19, BMI, smoking status, education level, working in the healthcare field, financial responsibility, and knowledge of COVID-19.

**Table 1:** Characteristics of the study sample grouped by reported depression versus no reported depression *Chi-square test; ^#^p-value based on the one-way ANOVA test.

Characteristics	Total sample (N%) 942 (100%)	No reported depression (N%) 698 (74%)	Reported depression (N%) 242 (26%)	p-value*
Age				
Less than 40	813(86)	585(72)	228(28)	0.000
More than 40	129(14)	113(81)	16(12)	
Sex				
Male	230(24)	186(89)	44(19)	0.009
Female	712(76)	514(72)	198(28)	
Marital status				
Single	522(56)	372(71)	150(29)	0.005
Married	389(41)	309(79)	80(21)	
Divorced or widowed	31(3)	19(61)	12(39)	
Nationality				
Non-Saudi	21(2.33)	15(71)	6(29)	0.760
Saudi	921(98)	685(74)	236(26)	
Diagnosed with Covid-19				
No	932(99)	691(74)	241(26)	0.254
Yes	10(1)	9(90)	1(10)	
BMI				
1 - Less than 18.5	216(23)	151(70)	65(30)	0.190
2 - Between (18.5-24.9)	339(36)	260(77)	79(23)	
3 - Between (25-29.9)	253(27)	194(77)	59(23)	
4 - 30 or above	134(14)	95(71)	39(29)	
Chronic diseases including chest diseases				
No	774(82)	597(77)	177(23)	0.000
Yes	168(18)	103(61)	65(39)	
Previous mental illnesses				
No	913(97)	684(75)	229(25)	0.017
Yes	29(3)	16(55)	13(45)	
Smoking status				
1 - Never smoked	847(90)	638(75)	209(25)	0.093
2 - Smoke's cigarettes or water pipe	46(5)	29(63)	17(37)	
3 - Ex-smoker	49(5)	33(67)	16(33)	
Employment status				
1 - Student	399(42)	272(68)	127(32)	0.000
2 - Public sector - private sector - free businesses	311(33)	253(81)	58(19)	
3 - Retired or not-working	232(25)	175(75)	57(24)	
Education levels				
Less than bachelor and more	214(23)	160(75)	54(25)	0.862
Bachelor and more	728(77)	540(74)	188(26)	
Standard of living level				
Limited	185(20)	118(64)	67(36)	0.000
Moderate	579(61)	435(75)	144(25)	
High	178(19)	147(83)	31(17)	
Working in healthcare field				
No	846(90)	630(74)	216(26)	0.742
Yes	96(10)	70(73)	26(27)	
Financially responsible				
No	692(73)	503(73)	189(27)	0.058
Yes	250(27)	197(79)	53(21)	
Reside				
Alone	21(2)	20(95)	1(5)	0.026
With one or more	921(98)	680(74)	241(26)	
Knowledge of COVID-19				
1 - No knowledge (score ≤3 points)	319(34)	225(71)	94(29)	0.114
2 - General knowledge (score 4 points)	511(54)	386(76)	125(24)	
3 - High knowledge (score ≥5 points)	112(12)	89(79)	23(21)	
Time spent focusing on the COVID-19				
<1 hour	699(74)	535(77)	164(23)	0.004
1-2 hours	166(18)	119(72)	47(28)	
≥3 hours	77(8)	46(60)	31(40)	

Table 2 shows the multi-logistic regression with several statistically significant factors that were associated with reported depression during the COVID-19 pandemic as follows: females 87% more compared to males, aged less than 40 are 57% more than participants aged more than 40, being divorced or widowed is 120% more than being single, participants with chronic diseases including chest diseases (160%), participants with previous mental illnesses are 150% more, being a student is 50% more than being a worker or retired, low income is 40% more and 60% more than moderate and high income, respectively, and time spent focusing on the COVID-19 is less than one hour daily.

**Table 2:** Multiple logistic regression analysis of factors associated with depression among Jazan population during Covid-19 pandemic *Chi-square test; ^#^p-value based on the one-way ANOVA test.

Characteristics	OR	95% CI		P-value
		Lower	Upper	
Age group (reference=less than or equal 40)				
More than 40	0.427	0.225	0.809	0.009
Gender (reference=male)				
Female	1.870	1.187	2.994	0.007
Social status (reference=single)				
Married	0.906	0.615	1.334	0.619
Divorced or widowed	2.181	1.012	5.012	0.049
Nationality (reference=non-Saudi)				
Saudi	0.906	0.356	2.419	0.880
Diagnosed with Covid-19 (reference=no)				
Yes	0.300	0.034	2.643	0.279
BMI (reference=less than 18.5)				
Between 18.5 and 24.9	0.817	0.554	1.227	0.332
Between 25 and 29.9	0.958	0.608	1.510	0.854
30 or above	1.288	0.767	2.164	0.338
Chronic diseases including chest diseases (reference=no)				
Yes	2.664	1.811	3.918	0.000
Previous mental illnesses (reference=no)				
Yes	2.509	1.111	5.666	0.027
Smoking status (reference=never smoked)				
Smoke's cigarettes or water pipe	1.914	0.920	3.981	0.082
Ex-smoker	1.435	0.706	2.913	0.317
Employment status (reference=student)				
Public sector–private sector–free businesses	0.549	0.335	0.899	0.017
Retired or not-working	0.599	0.393	0.913	0.017
Education levels (reference=less than bachelor)				
Bachelor and more	1.103	0.757	1.608	0.607
Standard of living level (reference=low)				
Moderate	0.617	0.421	0.903	0.013
High	0.379	0.222	0.647	0.001
Working in healthcare field (reference=no)				
Yes	1.433	0.867	2.368	0.160
Financially responsible (reference=no)				
Yes	1.001	0.628	1.592	0.998
Knowledge of COVID-19 (reference=no knowledge (score ≤3 points))				
General understanding (score 4 points)	0.783	0.561	1.094	0.153
High understanding (score ≥5 points)	0.642	0.382	1.081	0.096
Time spent focusing on the COVID-19 (reference≤1 hour)				
1–2 hours	1.302	0.868	1.951	0.201
≥3 hours	2.285	1.343	3.887	0.002

## Discussion

This study assessed the association of depression rates with the 2020 COVID-19 pandemic and the risk factors in the Jazan Region, KSA. Depression is one of the top 25 illnesses of global concern and is associated with increases in other diseases and suicide, making it crucial to address for policymakers [6]. Our study observed that the depression rate of participants was nearly 26%, which is in the mid-range of a previous systematic review of the association of the pandemic with depression rates, which reported rates as low as 14.6% to as high as 32.7% in countries as diverse as Denmark, the U.S., China, Nepal, Spain, Italy, and Iran [24]. Several other international studies reported rates of 14-18% in China, 18.7% in Spain, 32.7% in Italy, 23.6% in Turkey, and 25.4% in Denmark [11,13,25-29].

Our data revealed a statistically significant relationship between some factors and the increasing frequency of depressive symptoms among the participants. Compared to participants with no pre-existing conditions, those with chronic diseases had a 160% higher incidence of depressive symptoms. This finding is consistent with several international studies of the association of the COVID-19 pandemic with depression, reporting that patients with chronic diseases or multiple comorbidities are at an increased risk of psychological symptoms, including depression, possibly due to awareness of the severity of COVID-19 for this group [30-33]. Compared to those with no history of mental illness, participants with a history of diagnosed mental illness had a 150% higher incidence. This finding is also consistent with international studies that do have some mixed findings, as reported by Bell et al., in which one case-controlled study in the Netherlands found that although a history of mental illness predisposes individuals to a higher risk of depression, the rates did not increase in response to the pandemic [34,35]. However, the preponderance of studies reported results consistent with this study or higher rates of depression among those with a history of mental illness [36-40]. Increasing focus on the pandemic increased depression, whereby compared to those who focused on it less than one hour daily, those who focused on it three hours or more daily had a 130% higher incidence of depressive symptoms. This finding aligns with other international studies that have examined the association of consuming a lot of media coverage of the pandemic with the participants' mental state [41,42].

With media coverage now expanded to innumerable sources, there is a constant flow of information, much of which may be exaggerated or misleading, but nevertheless will have an impact on the mental health of those who consume large quantities of it [43-45]. While excessive focus on the pandemic is correlated with negative mental health effects, social media platforms represent a dual-edged sword. Chinese studies have shown that 80% of the population utilizes social media for news and in India, more than 90% obtain their medical information via the internet [9]. On one hand, the barrage of misinformation, conspiracy theories, and negative personal posts leads to increased frustration when trying to discriminate between sources [46]. On the other hand, the connectivity provided with the outside world during enforced quarantine can lessen the sense of isolation [46]. Additionally, during this pandemic, legitimate health authorities placed accurate information on these sites and users also created their own information networks to inform their contacts of resources, such as in India when there were shortages of oxygen and ventilators, while communication was made possible between family members and hospitalized COVID patients [46]. Finally, many studies, such as this one, have relied on social media platforms to conduct primary research.

Compared to single participants, those who were either divorced or widowed had a 120% higher incidence. Compared to males, female participants had an 87% higher incidence, while compared to those over 40, those under 40 had a 57% higher incidence. Other studies have also shown that females have demonstrated higher rates of depressive symptoms in response to COVID-19 [13,47-49], but the reasons for this are still speculative, such as the increased burden of care borne by females, hormonal influences, and brain reactivity that results in higher fear responses [50-52]. Many other studies have similarly documented that younger people have had higher rates of depressive symptoms, possibly due to their higher exposure to social media with its attendant constant coverage of the pandemic, and this group may be more impacted by lockdowns and social isolation [48,53-55]. Economic factors also played a role in that, compared to participants who were either working or retired, students had a 50% higher incidence of depressive symptoms, which is consistent with many other studies. A study from the Netherlands measured mood homeostasis before and during a stringent lockdown on college students and reported a decrease in the mood-elevating activities after the lockdown compared to before [56]. Multiple studies have confirmed the association of increased depressive symptoms and the COVID-19 pandemic among students for reasons of isolation, lack of social support, worry about missing school, and others [53,57-60]. Finally, our finding that income levels represent a risk since, compared to participants with a moderate or high income, those with a low income had a 40% and 60% higher incidence, respectively, is supported by other international studies [54,55].

The KSA had prepared for a pandemic after the MERS outbreak of 2012 and thus handled the COVID-19 pandemic in 2020 relatively well. However, the mental health aspects were not anticipated. In view of the increase in depressive symptoms accompanying the COVID-19 pandemic, it is wise to take this opportunity to plan mitigation measures both for the current as well as any future pandemics. High-risk groups included those with comorbidities, a history of mental illness, a habit of focusing on the pandemic, females, young people, students, and low-income participants. Strategies to address the needs of these groups should be sought from multiple sectors of society, especially institutions such as schools. The medical establishment should address mental health issues so that they are part of risk management along with the medical consequences of pandemics. Ozamiz-Etxebarria has suggested that academic support be enhanced to help alleviate some of the psychological effects on students and the young [31]. The provision of a central source of accurate information may alleviate some of the fear and depression that are fueled by sensationalist news and misinformation from the public media. Further research is needed to find the source of depressive symptoms in the higher risk groups and to measure the mental health impacts of the pandemic over the long term in the Jazan Region.

Limitations

Although this first study of depression associated with the COVID-19 pandemic is very rigorous, we must mention its limitations. First, this study should not be generalized to the entire population of the KSA as it is specific to the Jazan region, which may differ from the population as a whole. Second, the necessity of conducting this original research through an online survey automatically excludes those who do not participate in the applications through which it was distributed. Third, the use of a convenience sample may have inadvertently led to sampling bias, although we followed standard protocol to avoid such bias. Finally, the survey instrument cannot assign cause and effect to this cross-sectional study.

## Conclusions

In the Jazan region, the rate of depression during the COVID-19 pandemic was nearly 26%. In addition, there are several significant determinants associated with higher rates of depression, in descending order: history of chronic diseases, history of mental illness, excessive focus on the pandemic, being divorced or widowed, female gender, being under age 40, being a student, having a low income. This study clearly demonstrates a relationship between the COVID-19 pandemic and depression in the Jazan region. We suggest prioritizing mental health prior to the next pandemic, encouraging the medical establishment to plan to integrate mental health into the care provided to vulnerable populations, and a public information system to provide accurate information in the event of such a health crisis. We recommend further research into more detailed causal effects of depression and also longer-term studies to capture the changing mental health landscape. Additional suggestions are noted in the discussion in this paper.
